# Delayed but Dangerous: Chronic Encapsulated Expanding Hematoma as a Reversible Cause of Steroid-Resistant Extensive Edema Following Stereotactic Radiosurgery for Cerebral Arteriovenous Malformation

**DOI:** 10.7759/cureus.84035

**Published:** 2025-05-13

**Authors:** Prasert Iampreechakul, Wuttipong Tirakotai, Punjama Lertbutsayanukul, Samasuk Thammachantha, Mantana Dhanachai

**Affiliations:** 1 Department of Neurological Surgery, Neurological Institute of Thailand, Bangkok, THA; 2 Department of Neuroradiology, Neurological Institute of Thailand, Bangkok, THA; 3 Department of Pathology, Neurological Institute of Thailand, Bangkok, THA; 4 Department of Diagnostic and Therapeutic Radiology, Ramathibodi Hospital, Mahidol University, Bangkok, THA

**Keywords:** brain arteriovenous malformation, chronic encapsulated expanding hematoma, radiation necrosis, stereotactic radiosurgery, steroid-resistant edema, vasogenic edema

## Abstract

Chronic encapsulated expanding hematoma (CEEH) is a rare, delayed complication of stereotactic radiosurgery (SRS) for brain arteriovenous malformations (AVMs), often masquerading as radiation necrosis or tumor recurrence. We report the case of a 61-year-old man who developed progressive right hemiparesis and extensive steroid-resistant vasogenic edema 15 years after embolization and multisession SRS for a ruptured left parietal AVM, which had been previously confirmed as completely obliterated. Initial imaging showed a small enhancing lesion with surrounding edema, suspected to be radiation necrosis. Over the next two years, the lesion expanded with persistent edema despite corticosteroids and decompressive craniectomy. Follow-up MRI revealed classic hemorrhagic features, including a peripheral hypointense rim on T2*-GRE and SWI sequences, raising suspicion for CEEH. Surgical resection confirmed the diagnosis histopathologically and was followed by rapid clinical and radiological improvement. This case underscores the importance of considering CEEH in patients presenting with delayed symptoms and steroid-resistant edema after SRS, even when the lesion appears small. Early surgical recognition and intervention can be both diagnostic and therapeutic, preventing further morbidity.

## Introduction

Stereotactic radiosurgery (SRS) has become a widely accepted, minimally invasive treatment option for intracranial arteriovenous malformations (AVMs), particularly when lesions are located in deep or eloquent brain regions where surgical intervention poses a high risk. The therapeutic effect of SRS relies on radiation-induced endothelial injury, smooth muscle proliferation, and subsequent luminal thrombosis and fibrosis, leading to gradual obliteration of the AVM nidus, typically within two to five years after treatment [1]. Although SRS is generally considered safe and effective, delayed complications may arise, including radiation necrosis, cyst formation, and the less common entity known as chronic encapsulated expanding hematoma (CEEH) [2,3].

CEEH is a rare but clinically important long-term complication of SRS, typically occurring several years after complete angiographic obliteration of the AVM. Histologically, it consists of a hematoma encased within a fibrous capsule, often associated with progressive perilesional vasogenic edema and neurological symptoms. The proposed pathogenesis involves radiation-induced vascular fragility, aberrant neovascularization, and repeated microhemorrhages. Notably, recent studies have identified high levels of vascular endothelial growth factor (VEGF) within the hematoma capsule, supporting the role of angiogenic dysregulation in its formation [4-6]. Radiologically, CEEH may appear as a heterogeneous mass with a characteristic peripheral hypointense rim and extensive surrounding edema, often mimicking other post-radiosurgical entities such as radiation necrosis or tumor recurrence [7].

Recent retrospective cohort studies estimate the incidence of CEEH at approximately 0.28% to 0.6% among AVM patients treated with SRS, with latency periods extending from five to more than 20 years [4,8]. Surgical excision of the hematoma and its capsule typically results in symptomatic relief and resolution of edema, especially in patients with progressive symptoms. In contrast, conservative observation may be considered for smaller, asymptomatic lesions located in deep or high-risk regions [4,5,9]. Despite its potential severity, CEEH remains underrecognized, in part due to its delayed onset and radiological overlap with other post-treatment complications.

Herein, we describe a rare case of CEEH presenting 15 years after embolization and multisession SRS for a ruptured left parietal AVM. The lesion manifested with progressive hemispheric vasogenic edema that was resistant to corticosteroid therapy and initially misinterpreted as radiation necrosis. This case highlights the importance of maintaining long-term vigilance for CEEH and considering it in the differential diagnosis of delayed neurological decline following AVM radiosurgery.

## Case presentation

A 61-year-old man was admitted to a local hospital with progressive right hemiparesis for five days. He had a history of a seizure two months prior, which began as a focal seizure in the right forearm, progressed to a generalized seizure, and was followed by a brief loss of consciousness lasting approximately two minutes. He did not seek medical attention at that time and was discharged after two days. He subsequently developed intermittent hiccups and alternating muscle twitching in both legs, prompting readmission. A cranial computed tomography (CT) was performed, and the patient was informed that he had a brain tumor with surrounding edema requiring surgery. He declined surgical intervention and was referred to our institute.

**Figure 1:**
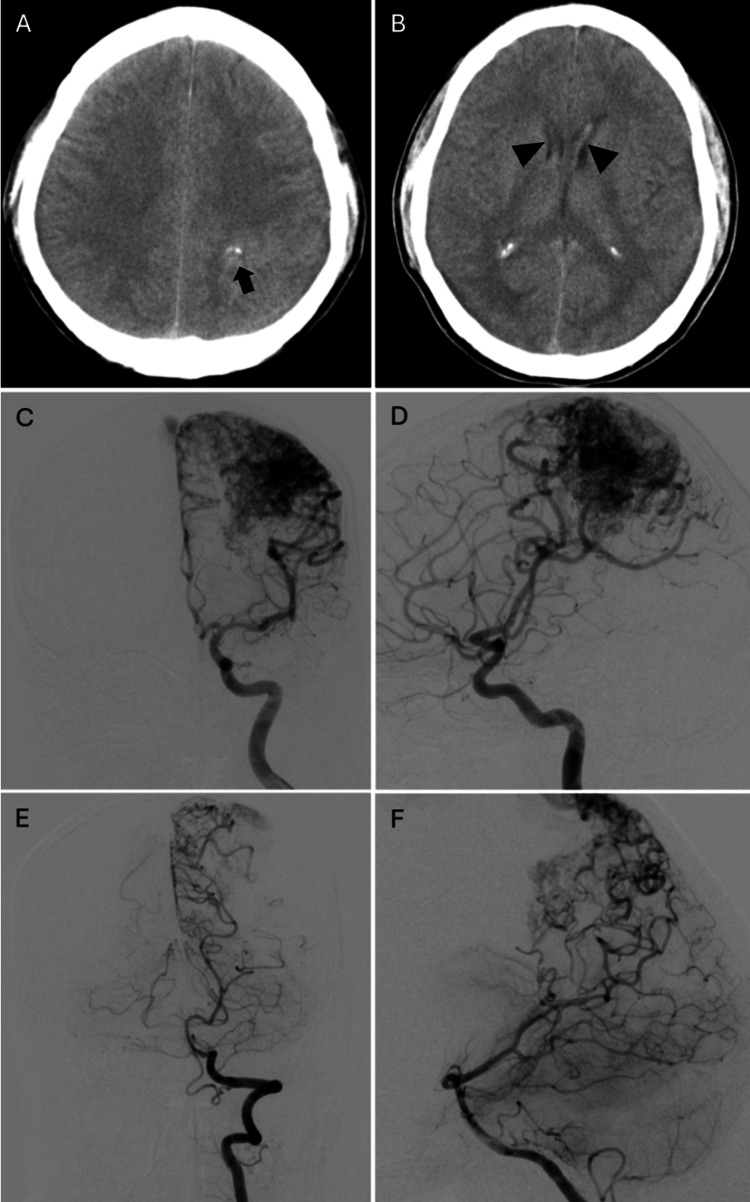
Initial cranial CT scan and cerebral angiography CT: computed tomography; AP: anteroposterior; AVM: arteriovenous malformation (A-B) Sequential axial CT images demonstrate a superficially located, ill-defined, mildly hyperdense lesion in the left parietal lobe with internal calcifications (arrow) and associated intraventricular hemorrhage (arrowheads) involving both lateral ventricles. (C) AP and (D) lateral views of a left internal carotid artery injection and (E) AP and (F) lateral views of a left vertebral artery injection reveal a large AVM located in the left parietal region

His past medical history revealed that in June 2008, he experienced a sudden, severe headache. A cranial CT scan demonstrated a ruptured AVM in the left parietal region. Initial cerebral angiography revealed a large AVM extending from the periventricular region to the cortical surface of the left parietal lobe, corresponding to a Spetzler-Martin Grade III lesion (Figure 1). The patient underwent multisession transarterial embolization using N-butyl cyanoacrylate (NBCA) at our institute.

**Figure 2:**
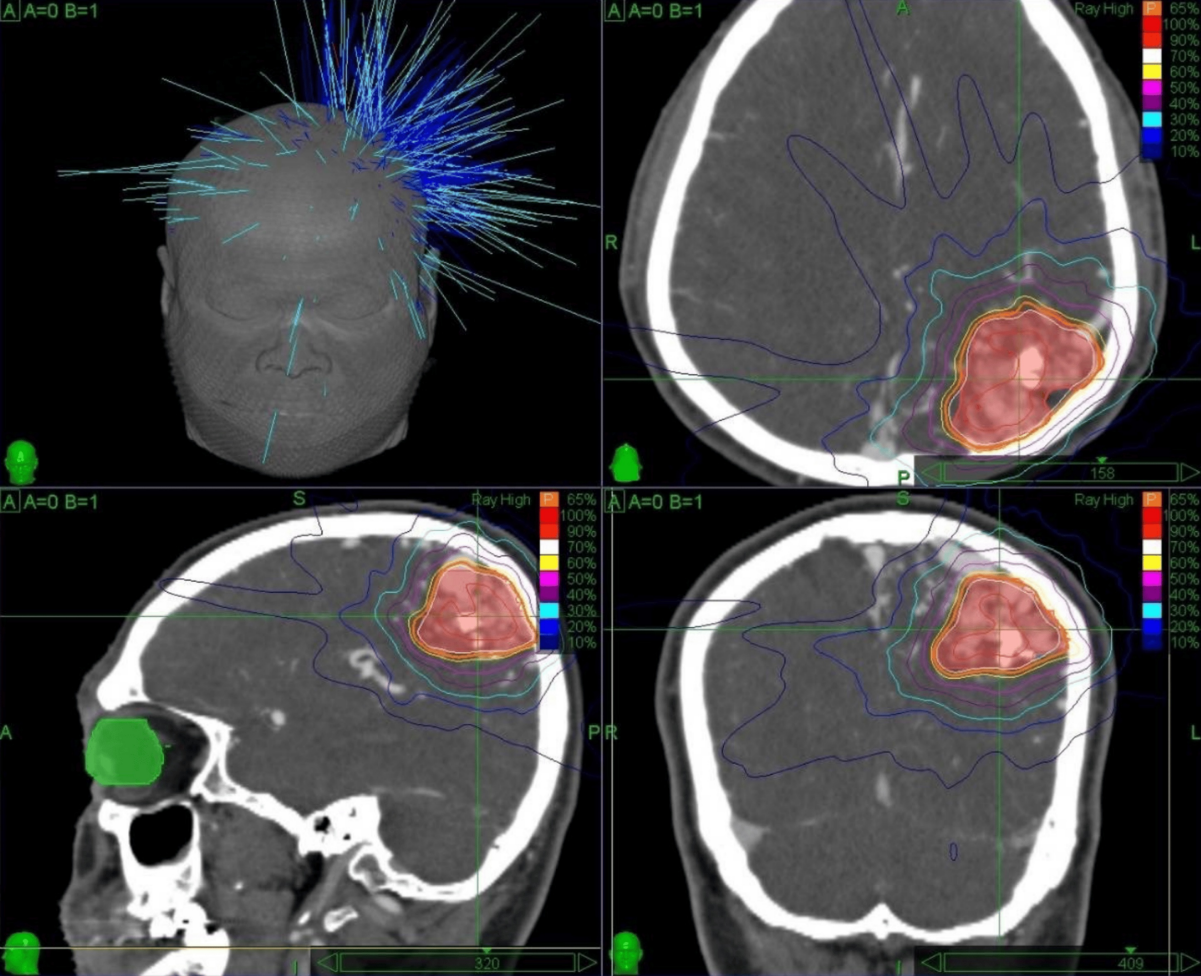
CyberKnife dose distribution plan for left parietal AVM AVM: arteriovenous malformation Dose distribution map of CyberKnife stereotactic radiosurgery administered for a left parietal AVM with a total nidus volume of 26.8 cc. A total dose of 30 Gy was delivered in five fractions (5.5 Gy per fraction), prescribed to the 65% isodose line, over the course of one week

In December 2009, the patient underwent SRS at another university hospital. The residual AVM, with a total volume of 28.192 cc, was treated using a CyberKnife system with a five-fraction regimen (Figure 2). The prescribed dose was 27.5 Gy, with a per-fraction dose of 5.5 Gy. Treatment was delivered at a 65% isodose line with a conformity index of 1.14.

**Figure 3:**
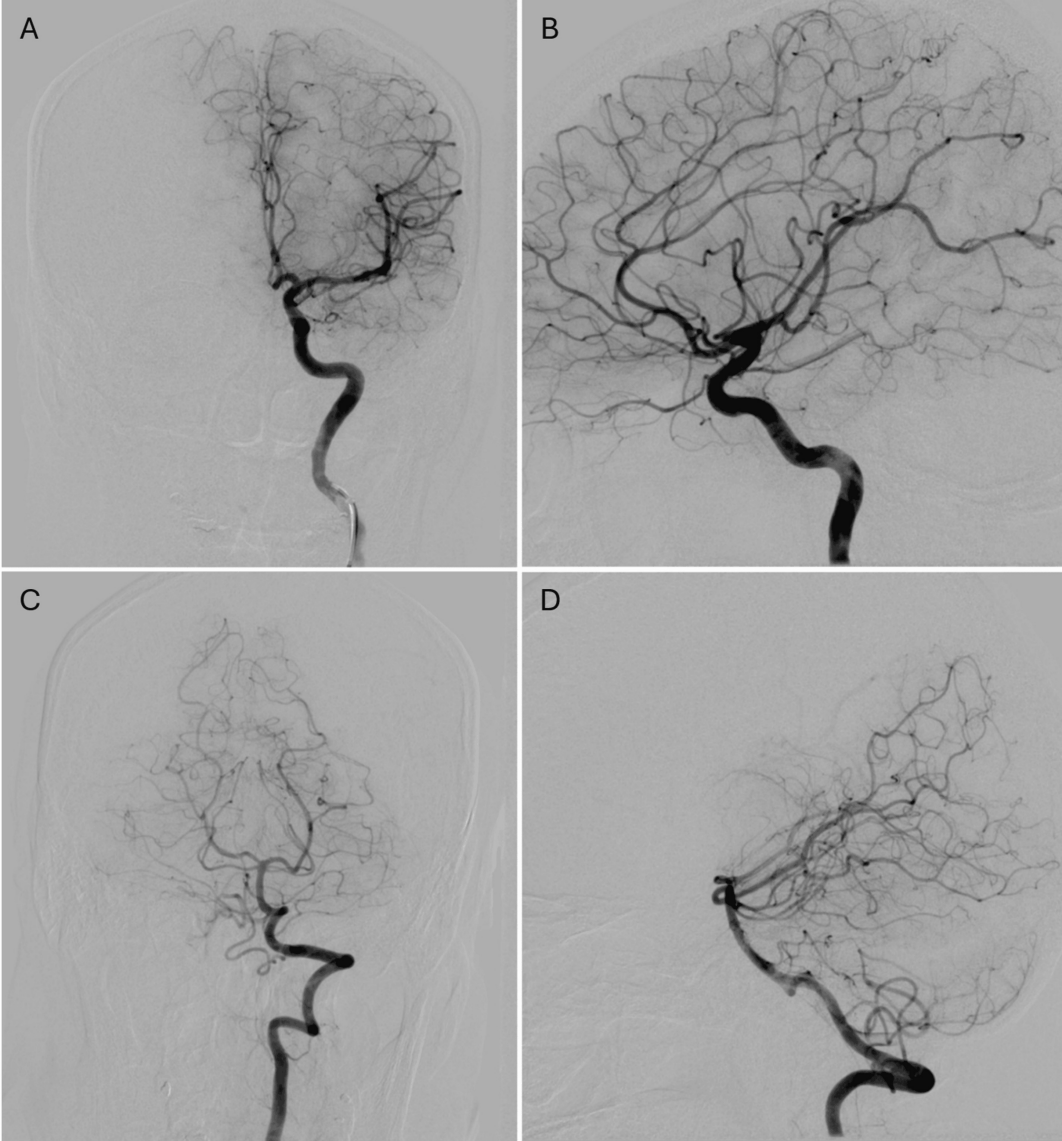
Cerebral angiography obtained three years after stereotactic radiosurgery AVM: arteriovenous malformation; AP: anteroposterior (A) AP and (B) lateral views of a left internal carotid artery injection, along with (C) AP and (D) lateral views of a left vertebral artery injection, confirm complete obliteration of the previously identified left parietal AVM

**Figure 4:**
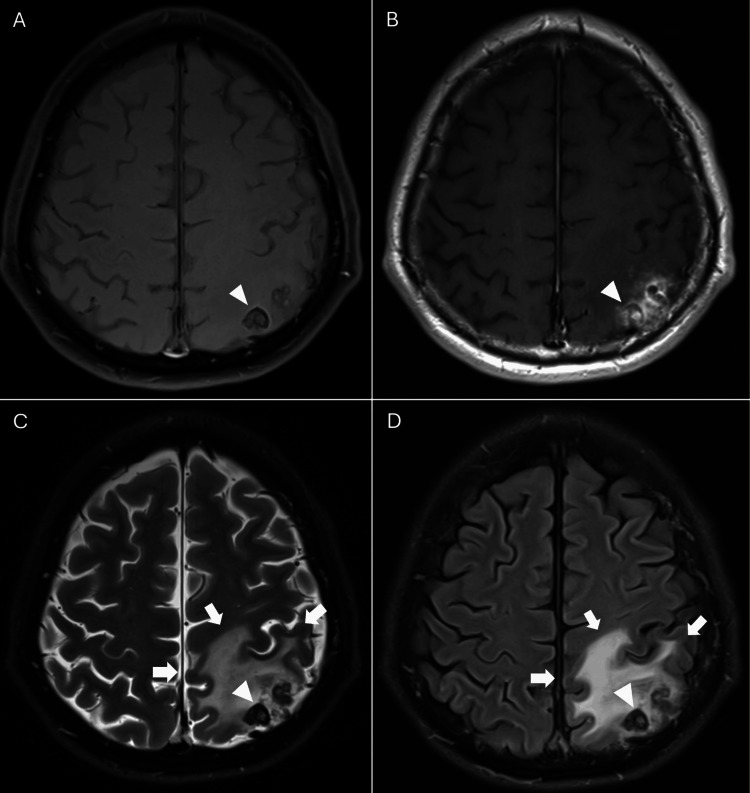
Cranial MRI obtained 13 years after stereotactic radiosurgery MRI: magnetic resonance imaging; FLAIR: fluid-attenuated inversion recovery Axial (A) T1-weighted image without contrast, (B) T1-weighted image with contrast, (C) T2-weighted image, and (D) FLAIR image reveal a small, round, heterogeneous lesion (with mixed iso-, hypo-, and mildly hyperintense signals) exhibiting a peripheral low-signal rim (arrowheads) and patchy internal and perilesional enhancement. Surrounding vasogenic edema (arrows) is noted, predominantly involving the left superior parietal lobule

Follow-up cerebral angiography in January 2013 confirmed complete obliteration of the AVM (Figure 3). The patient remained asymptomatic and underwent clinical follow-up every two years. In November 2022, magnetic resonance angiography (MRA) showed no recurrence of AVM. However, MR imaging (MRI) revealed a small, round, heterogeneous lesion in the left superior parietal lobule, with mixed iso-, hypo-, and mildly hyperintense signals and a peripheral low-signal rim. Patchy enhancement was noted within and around the lesion, along with surrounding edema (Figure 4). The lesion was suspected to represent post-radiosurgical change. As the patient was asymptomatic, observation without treatment was recommended.

**Figure 5:**
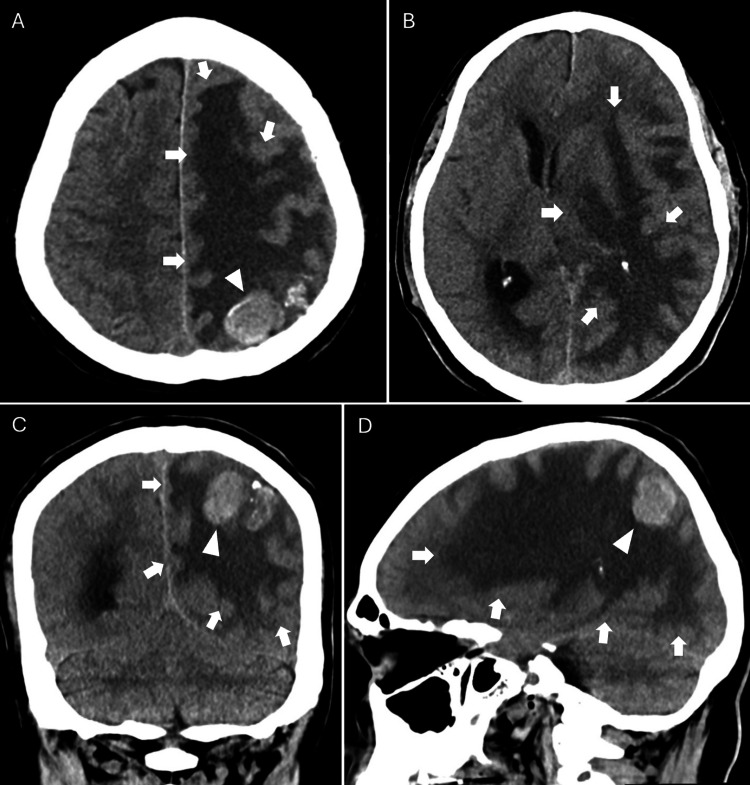
Cranial CT obtained 15 years after stereotactic radiosurgery CT: computed tomography (A-B) Axial, (C) coronal, and (D) sagittal views demonstrate a hyperdense, round lesion (arrowheads) at the cortical surface of the left parietal lobe. Extensive, frond-like hypodensity consistent with progressive vasogenic edema (arrows) is observed, involving the left frontal, parietal, and occipital lobes, accompanied by a rightward midline shift

In January 2025, the patient presented to our institute with new-onset drowsiness, psychomotor slowing, and right-sided hemiparesis (motor grade 1/5). Cranial CT revealed a hyperdense, round lesion at the cortical surface of the left parietal lobe. Extensive frond-like hypodensity consistent with vasogenic edema was noted, involving the left frontal, parietal, and occipital lobes, as well as the insular white matter, internal and external capsules, and splenium. A midline shift of 7 mm and evidence of left uncal herniation were also present (Figure 5). At the time of decompressive craniectomy, the primary surgical objective was to urgently relieve life-threatening intracranial pressure resulting from the extensive edema and mass effect. The exact nature of the left parietal lesion remained undetermined. Given the diagnostic uncertainty and the severity of surrounding edema, direct resection or biopsy of the lesion during the initial surgery was deferred to avoid increased surgical risk. Instead, a staged approach was adopted, prioritizing clinical stabilization and follow-up imaging to better define the lesion prior to definitive intervention.

**Figure 6:**
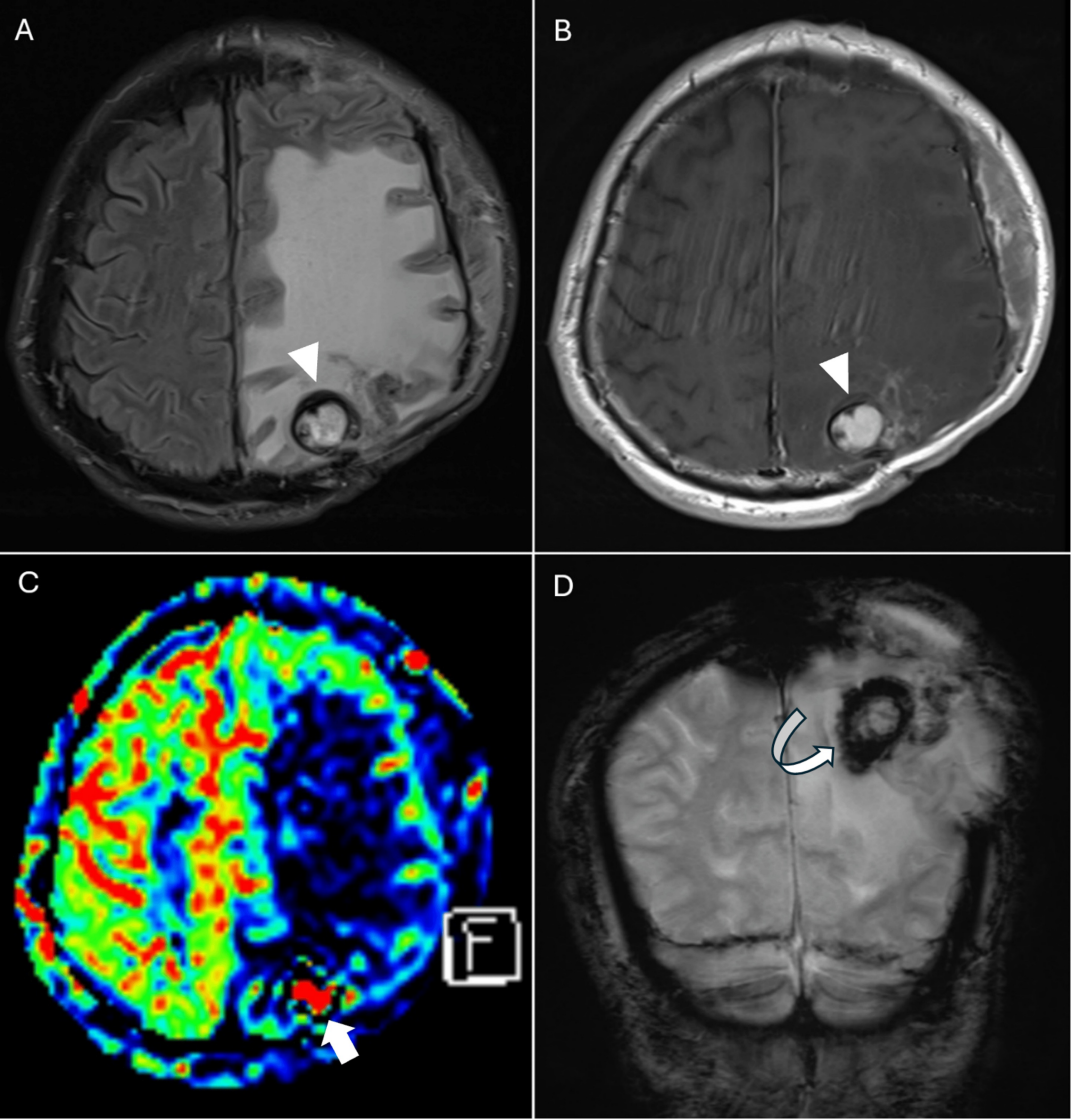
Cranial MRI obtained 15 years after stereotactic radiosurgery and two weeks after decompressive craniectomy and intravenous steroid treatment MRI: magnetic resonance imaging (A) Axial T2-weighted image and (B) axial T1-weighted image with contrast show enlargement of the left parietal enhancing lesion with a peripheral low-signal rim (arrowheads) and persistent progressive vasogenic edema. (C) Axial arterial spin labeling (ASL) perfusion imaging demonstrates focal hyperperfusion (arrow) within the lesion and surrounding hypoperfusion corresponding to the edematous areas. (D) Coronal T2*-weighted gradient-echo (GRE) image reveals a low-signal (dark) rim (curve arrow) around the lesion, consistent with hemosiderin deposition or prior hemorrhage

Two weeks after decompressive surgery, MRI showed enlargement of the left parietal enhancing lesion with a peripheral low-signal rim. Susceptibility-weighted imaging (SWI) and T2*-weighted gradient-echo (GRE) sequences revealed a dark rim suggestive of hemosiderin deposition, raising suspicion for CEEH. The vasogenic edema remained extensive and unresponsive to steroids, and the patient continued to have the right hemiparesis with no improvement, remaining at motor grade 1/5 (Figure 6). Surgical resection of the lesion was performed the following day.

**Figure 7:**
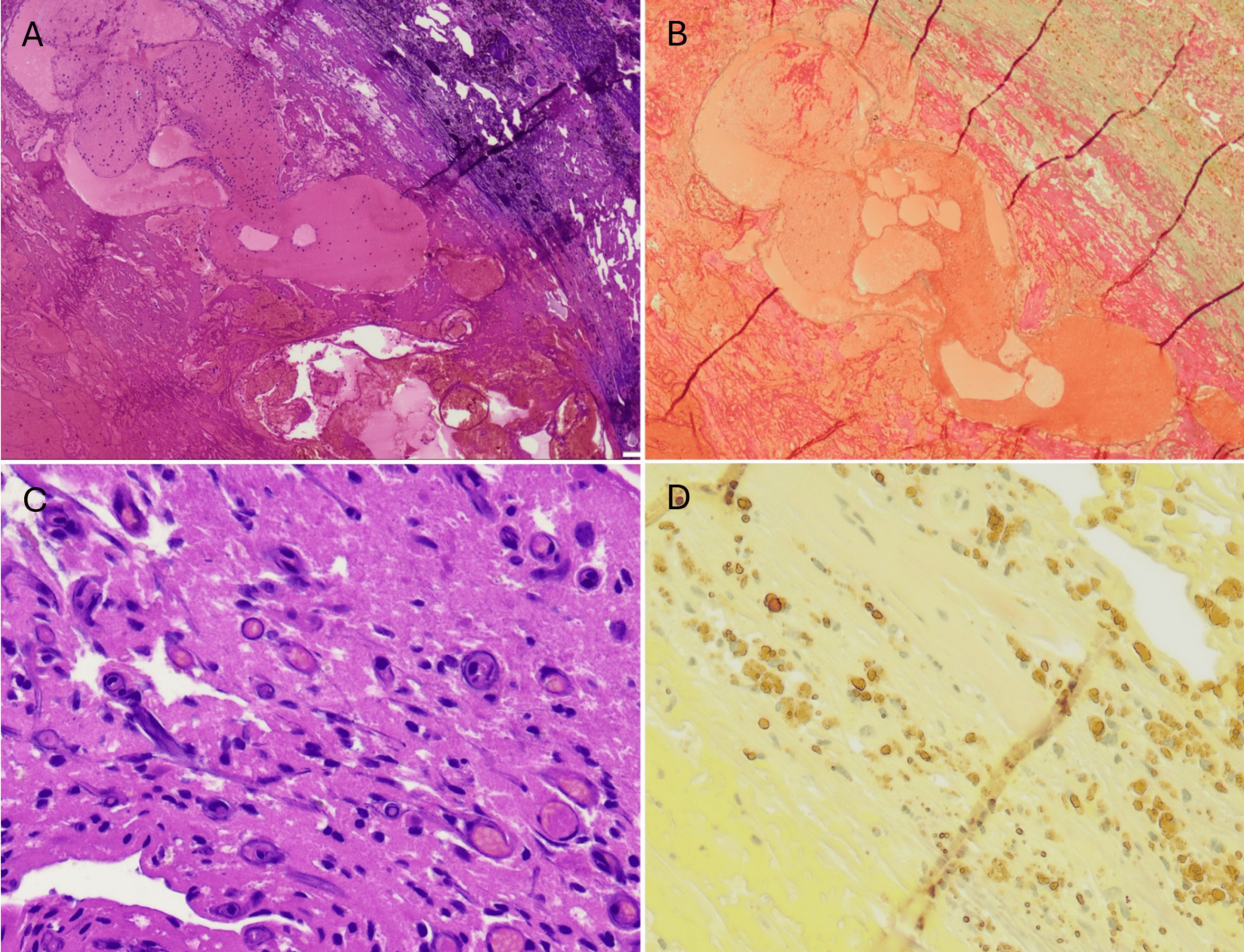
Histopathological findings H&E: hematoxylin and eosin (A) Photomicrograph demonstrates central hemorrhagic necrosis with fibrin deposition and organizing thrombus, surrounded by peripheral fibrosis and vascular ferrugination (H&E stain, ×40 magnification). (B) Masson’s trichrome stain highlights dense peripheral fibrosis (×40 magnification). (C) Higher-magnification H&E staining reveals numerous newly formed capillaries adjacent to the lesion (×200 magnification). (D) Elastic Van Gieson stain shows abundant hemosiderin pigment deposition within the lesion (×200 magnification), consistent with chronic hemorrhage

Histopathological examination revealed central hemorrhagic necrosis with fibrin deposition and organizing thrombus, surrounded by peripheral fibrosis and vascular ferrugination. Masson’s trichrome stain confirmed dense fibrosis, while the Elastic Van Gieson stain demonstrated hemosiderin deposits. Numerous newly formed capillaries were seen adjacent to the lesion, consistent with CEEH (Figure 7). No definitive histological evidence of radiation-induced changes, such as vascular hyalinization, fibrinoid necrosis, or parenchymal rarefaction, was identified in the resected specimen.

**Figure 8:**
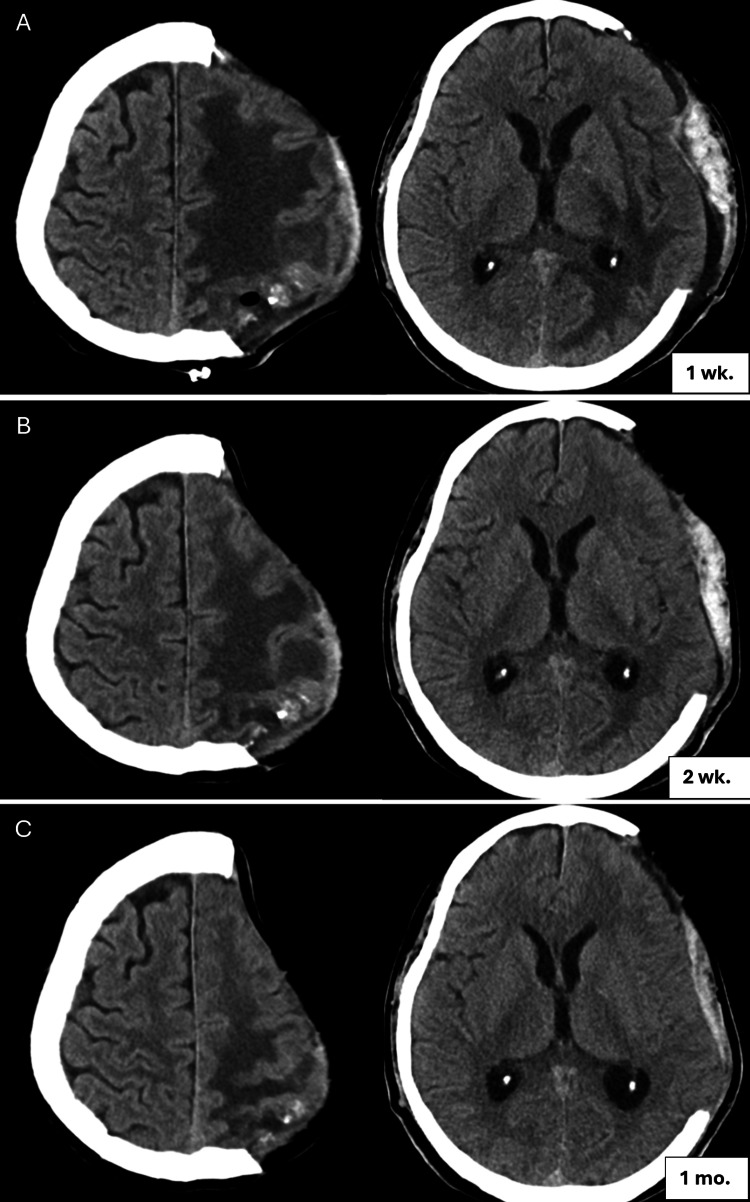
Follow-up cranial CT after surgical removal of CEEH CT: computed tomography; CEEH: chronic encapsulated expanding hematoma Axial views obtained at (A) one week, (B) two weeks, and (C) one month postoperatively demonstrate rapid reduction of vasogenic edema in the left cerebral hemisphere following surgical resection of the CEEH

The postoperative course was uneventful. Follow-up cranial CT scans at one week, two weeks, and one month after surgery demonstrated rapid resolution of vasogenic edema (Figure 8). The patient’s right-sided weakness improved significantly, and by two months postoperatively, he was able to ambulate with support.

## Discussion

Brain AVMs are complex vascular anomalies that pose a lifelong risk of hemorrhage, especially in pediatric patients whose cumulative risk increases over time. Multimodal treatment strategies are commonly employed, with endovascular embolization often used as a pre-treatment to reduce nidus size or flow prior to SRS, thereby enhancing the safety and efficacy of radiation delivery. Long-term follow-up is essential, as AVM obliteration can take several years post-SRS, and delayed complications such as radiation necrosis (RN), cyst formation, or even recurrence may occur. Serial imaging, including MRI and confirmatory angiography, remains the standard for monitoring treatment response and detecting late adverse events or AVM regrowth, which underscores the importance of sustained surveillance even after apparent radiographic cure [10-13].

RN is a well-recognized delayed complication following SRS for brain AVMs, typically emerging between six and 36 months post-treatment. It results from radiation-induced vascular damage, leading to blood-brain barrier disruption, inflammatory cascade activation, and progressive brain tissue necrosis. The incidence of radiologic radiation-induced changes, including T2/fluid-attenuated inversion recovery (FLAIR) hyperintensity and edema, ranges from 30% to 40% of treated patients, while symptomatic RN occurs in approximately 7-9%, with permanent deficits in a smaller subset (1-4%) [14-17]. Predictive factors include larger AVM volume, eloquent brain location, and higher radiation dose or volume, notably when the 12 Gy volume (V12) exceeds 10 cm³, which has been associated with an increased risk of symptomatic RN [16,18]. While most asymptomatic RN cases are managed conservatively, symptomatic RN typically responds to corticosteroids, though resistant cases may benefit from bevacizumab or surgical resection in select scenarios [15]. Accurate diagnosis remains challenging due to overlap with recurrent AVM or CEEH, highlighting the value of multimodal imaging and the need for long-term surveillance after SRS.

Chronic encapsulated intracerebral hematoma (CEIH) is a rare condition characterized by a slowly enlarging, fibrous capsule-enclosed hematoma that may mimic a brain tumor. Unlike typical resolving hematomas, CEIH persists or grows due to recurrent microbleeds and capsule formation. MRI is useful for diagnosis, often showing a peripheral low-signal rim on T2-weighted images. Histologically, CEIH shows organized hemorrhage, fibrosis, and hemosiderin deposition. Early recognition is important to avoid misdiagnosis and guide appropriate surgical management [19].

In a single-center retrospective study of 950 patients treated with Gamma Knife SRS for intracranial AVMs over a 30-year period, Abou-Al-Shaar et al. [8] identified six cases (0.6%) of CEEHs, yielding an incidence rate of 0.0045 events per person-year. The onset of these lesions ranged from 5.5 to 20 years after treatment and was not associated with AVM recurrence, as angiographic obliteration had been confirmed in all cases. Imaging typically revealed a slowly enlarging, well-circumscribed mass at the prior AVM site with heterogeneous signal, peripheral enhancement, and surrounding edema. Histopathological analysis demonstrated a fibrotic capsule with immature neovascularization, hemosiderin deposition, and chronic inflammation, consistent with radiation-induced microvascular injury. All patients underwent surgical resection, with favorable neurological outcomes in all but one case that experienced a postoperative infection. These findings highlight the need for long-term surveillance after SRS and reinforce the importance of considering CEEH in the differential diagnosis of delayed mass lesions following AVM obliteration.

A recent international multicenter study of 5430 patients who underwent SRS for intracranial AVMs reported a crude incidence of CEEH of 0.28%, with a median latency of 106 months after treatment. CEEHs typically arose in lobar regions and presented with headaches, seizures, or neurological deficits, although some were discovered incidentally. Histopathology consistently revealed chronic hemorrhage, fibrosis, neovascularization, and hemosiderin deposition, with strong VEGF expression in the hematoma capsule, supporting the hypothesis of radiation-induced angiogenic proliferation as a key mechanism. Surgical resection yielded favorable outcomes in most symptomatic cases, while conservative management was reserved for stable or deep-seated lesions. These findings underscore the importance of long-term surveillance in post-SRS patients and the need to consider CEEH as a distinct entity from RN when evaluating delayed mass lesions [4].

Differentiating CEEH from RN can be challenging, but certain MRI features are helpful. CEEH typically presents as a well-circumscribed, gradually enlarging lesion with a peripheral hypointense rim on T2*-weighted GRE and SWI, which reflects hemosiderin from chronic hemorrhage. It often shows heterogeneous internal signal intensity and patchy enhancement. In contrast, RN more commonly demonstrates a "Swiss cheese" or "soap bubble" pattern on T1 post-contrast images, with central necrosis, irregular ring enhancement, and surrounding FLAIR hyperintensity [15]. Both conditions may lead to steroid-resistant edema, but CEEH frequently displays blooming artifacts on SWI and progressive lesion enlargement. Arterial spin labeling (ASL) may help differentiate the two, as CEEH often shows focal hyperperfusion due to neovascularization, while RN typically exhibits hypoperfusion.

This case illustrates a delayed presentation of CEEH that was initially misinterpreted as RN due to the small lesion size, mild edema, and absence of symptoms at the time of detection. Over the next two years, the lesion enlarged and was accompanied by extensive vasogenic edema, which did not respond to corticosteroids or decompressive craniectomy. Subsequent MRI revealed imaging features typical of CEEH. Surgical resection confirmed the diagnosis, with histopathology demonstrating fibrous encapsulation, chronic hemorrhage, neovascularization, and hemosiderin deposition. The patient experienced rapid neurological and radiological improvement following surgery.

The absence of acute symptoms and common risk factors for spontaneous hemorrhage, along with the lesion’s location at the prior AVM nidus, supports a radiation-induced origin. Although the resected specimen did not show definitive histologic signs of radiation injury, such as fibrinoid necrosis or vessel hyalinization, the chronic hemorrhagic and fibrotic features were consistent with delayed radiation-related changes. Similar findings have been reported in other cases that occurred several years after SRS, particularly in irradiated brain regions [4,8].

This case reinforces the importance of early recognition of CEEH. Even in asymptomatic patients, the appearance of a peripheral hypointense rim, blooming artifacts, and gradual lesion enlargement on serial imaging should raise suspicion. Rather than waiting for conservative treatments to fail or severe symptoms to emerge, earlier surgical removal may help prevent worsening edema, herniation, or prolonged disability. Timely diagnosis and intervention can provide both therapeutic benefit and histopathological confirmation.

The mechanism of extensive vasogenic edema in CEEH is believed to involve a combination of persistent blood-brain barrier (BBB) disruption, chronic inflammatory signaling, and recurrent microhemorrhage from fragile neovascularized vessels within the hematoma capsule. As blood degradation products such as thrombin, fibrin, and hemosiderin accumulate, they stimulate local astrocytic and microglial activation, perpetuating the release of cytokines and increasing capillary permeability [5,19]. This cascade contributes to sustained vasogenic edema that often extends far beyond the lesion itself. In some cases, particularly when the edema is longstanding and extensive, corticosteroids may fail to reduce swelling due to ongoing hemorrhagic and inflammatory stimuli that continue to disrupt the BBB [5]. In our patient, progressive hemispheric edema showed no response to high-dose corticosteroids or decompressive craniectomy. However, after surgical removal of the CEEH, there was a rapid and marked resolution of the edema, accompanied by significant neurological improvement. This supports prior findings that the hematoma capsule itself serves as the active source of edema, through continuous vascular leakage and inflammation [4,8]. Therefore, timely surgical excision is not only diagnostic but also therapeutically effective in relieving mass effect and halting the edema-promoting process.

## Conclusions

CEEH is a rare but clinically important late complication of SRS for cerebral AVMs. This case demonstrates several notable features, including a long latency of 15 years after SRS, a small lesion associated with extensive hemispheric vasogenic edema, and failure to respond to corticosteroid therapy and decompressive craniectomy. The initial diagnosis of radiation necrosis was later revised following surgical resection, which revealed a CEEH and led to rapid clinical and radiological improvement. Clinicians should maintain a high index of suspicion for CEEH in patients with delayed neurological deterioration and steroid-resistant edema after SRS. Timely surgical intervention can provide both diagnostic confirmation and effective symptom relief.
